# ICRF-159 enhancement of radiation response in combined modality therapies. II. Differential responses of tumour and normal tissues.

**DOI:** 10.1038/bjc.1979.96

**Published:** 1979-05

**Authors:** C. J. Kovacs, M. J. Evans, D. R. Burholt, L. L. Schenken

## Abstract

The combined effect of the chemotherapeutic agent ICRF-159 and radiation on the proliferative status of tumor/normal systems has been evaluated using the Lewis lung tumour in BDF1 mice. We have previously shown that a 25 mg/kg dose of ICRF-159, given at 3h intervals X4 before irradiation, significantly enhanced tumour growth retardation relative to a single dose of 100 mg/kg before irradiation. Whilst both single and fractionated drug treatments produced a transient inhibition of cell proliferation, comparisons of the temporal recovery from the antiproliferative effect of radiation in both tumour and intestinal epithelium suggested that single acute doses of ICRF-159 fail to potentiate the radiation response of either tissue. Protracted drug administration before irradiation, however, markedly decreases the post-radiation proliferative recovery of the tumour, without significantly altering intestinal recovery. The data suggest that both drug concentration and/or exposure time determine the interactions seen with combined modes.


*Br. J. Cancer* (1979) **39**, 524

### ICRF-159 ENHANCEMENT OF RADIATION RESPONSE IN COMBINED MODALITY THERAPIES. II. DIFFERENTIAL RESPONSES OF TUMOUR AND NORMAL TISSUES

C. J. KOVACS, M. J. EVANS, D. R. BURHOLT AND L. L. SCHENKEN

*From the Cancer Research Unit, Division of Radiation Oncology, Clinical Radiation Therapy Research Center, Allegheny General Hospital, Pittsburgh, PA 15212, U.S.A.*

Received 6 November 1978 Accepted 19 January 1979

**Summary.—The combined effect of the chemotherapeutic agent ICRF-159 and radiation on the proliferative status of tumour/normal systems has been evaluated using the Lewis lung tumour in $BDF_1$ mice. We have previously shown that a 25 mg/kg dose of ICRF-159, given at 3h intervals ×4 before irradiation, significantly enhanced tumour growth retardation relative to a single dose of 100 mg/kg before irradiation. Whilst both single and fractionated drug treatments produced a transient inhibition of cell proliferation, comparisons of the temporal recovery from the antiproliferative effect of radiation in both tumour and intestinal epithelium suggested that single acute doses of ICRF-159 fail to potentiate the radiation response of either tissue. Protracted drug administration before irradiation, however, markedly decreases the post-radiation proliferative recovery of the tumour, without significantly altering intestinal recovery. The data suggest that both drug concentration and/or exposure time determine the interactions seen with combined modes.**

ENHANCED efficacy of radiotherapy when combined with the drug ICRF-159 has been reported for both experimental tumours (Hellmann & Murken, 1974; Norpoth *et al.*, 1974; Peters, 1975) and in clinical trials (Bellet *et al.*, 1973; Ryall *et al.*, 1974). In the preceding report (Kovacs *et al.*, 1979), low-dose protracted drug pretreatment was found to be more effective than an acute dose for potentiating tumour-directed radiotherapy.

Relatively little information exists with respect to the cellular action of ICRF-159 on normal dividing tissues. Gralla *et al.* (1974) have reported reversible marrow and intestinal toxicity, which were both dose- and schedule-dependent. Most probably, optimal use of ICRF-159 in combination with radiation depends on the time/dose effects of each modality on both tumour cells and critical host organs. This is particularly true when the tumour-directed radiation field includes a substantial portion of a dose-limiting normal tissue.

The present report presents the differential response of the Lewis lung tumour and the underlying intestinal epithelium of the host after combined treatment with ICRF-159 and regional irradiation of the tumour.

### MATERIALS AND METHODS

*Tumour growth.*—Male $BDF_1$ mice obtained from Jackson Laboratories (Bar Harbor, ME) were inoculated s.c. in the back with $10^6$ cells of a single-cell suspension prepared from stock Lewis lung carcinoma (LL), originally obtained from Linda Simpson-Herren, Southern Research Institute, Birmingham, AL. The LL is routinely maintained by s.c. transplantation into $BDF_1$ males. The mice were maintained under a 12 h lighting schedule, and Purina Lab Chow and water supplied *ad libitum.*

*ICRF*-159.—ICRF-159 (NSC 129943) was supplied by Dr H. B. Wood, Drug Synthesis and Chemistry Branch, DCT, National Cancer Institute. The drug was finely suspended in a sterile solution of 0·5% (w/v) carboxymethylcellulose–saline (CMC–saline) and the suspension stirred for 30 min before injection. ICRF-159 was injected i.p. so that the volume of each injection was 0·01 ml/g body wt.

*Radiation*.—Irradiation was performed with a General Electric Maxitron 300. The physical factors were 275kVcp, 20 mA, H.V.L.=1·8 mmCu and TSD of 31 cm. Animals were positioned in leucite containers in such a way that the parts of the animal anterior to the xyphoid process, the femurs and the tail were shielded with lead. Animals were irradiated to a total dose of 600 rad with an exposure rate of 80 rad/min.

*$^3$H-Thymidine incorporation into DNA*.—At various times during and after treatment, 4 animals from each treatment group were injected with 50 $\mu$Ci of $^3$H-methyl-TdR (sp. act. 0·36 Ci/mmol, Schwarz/Mann, Orangeburg, NY). Thirty minutes later the animals were killed and the tumours and jejunal segments (4 cm) removed. The jejunum was opened and thoroughly rinsed in iced saline to remove material in the lumen. At the same time, tumours were dissected and one cross-sectional sample placed in 10% buffered formalin for histological analyses. The remaining tumour tissue and jejunum were then blotted on filter paper, weighed and placed in Carnoy's fixative for 24 h to remove unincorporated label. The weighed, fixed tissues were rinsed in 60% ethanol, solubilized in Soluene (Packard Inst. Corp.) and counted in a liquid scintillation spectrometer. $^3$H-TdR incorporation (d/min/mg wet weight) was determined for an estimate of the proliferative activity of the tissue (Hagemann *et al*., 1971; Schenken, 1976).

*Tumour-cell kinetics*.—For determination of $^3$H-TdR labelling index (LI) and mitotic index (MI), the fixed tumour samples were embedded in Paraplast, sectioned at 4 $\mu$m, and the sections stained by the Feulgen method. Autoradiographs were prepared with Kodak NTB-2 emulsion and the slides exposed at 4°C for 14 days. Slides were developed with Kodak D-19, fixed and the LI and MI determined microscopically from randomly selected fields (4000 cells/tumour).

### RESULTS

### *Effect of ICRF-159 and regional abdominal radiation on jejunal proliferative activity*

During fractionated (25 mg/kg 3-hourly ×4) as well as acute (100 mg/kg) treatment with ICRF-159, proliferative activity (d/min/mg) in the jejunum was depressed, with a rapid return to control levels before 24 h after treatment (Fig. 1). The proliferative nadirs are similar in magnitude. Whilst fractionated drug treatment extended ($\simeq$30 h) the depression of proliferative activity compared to acute drug treatment ($\simeq$18 h), the recovery to control levels of proliferation was accomplished in ~21 h from the last druge xposure for each treatment. This recovery to control levels was followed by an overshoot (~150% control), reaching a maximum at 36 h for fractionated and 48 h for acute treatments.

FIG. 1.—Proliferative response of the jejunal epithelium to acute and fractionated ICRF-159. (○——○), 100 mg/kg ICRF-159 at $t=0$; (●——●), 25 mg/kg 3-hourly × 4 from −12 h. Each point represents the mean d/min/mg ± s.e. for 4 animals.

The response of the jejunum to 600 rad abdominal X-irradiation and ICRF-159–radiation combinations is presented in Fig. 2. After 600 rad alone, a rapid depression in proliferative activity occurred. $^3$H-TdR incorporation returned to control levels 36–48 h after irradiation, with a maximum overshoot of incorporation at 48 h. Neither an acute dose of 100 mg/kg ICRF-159 (Fig. 2a) 5 min before irradiation, or a fractionated pretreatment with ICRF-159 at a dose of 25 mg/kg 3-hourly ×4 (Fig. 2b) before irradiation altered the initial jejunal response to 600 rad. Retardation of the overshoot peak (48 *vs* 72 h) was found for both drug+radiation

FIG. 2 (a).—The effect of combined acute ICRF-159 and 600 rad on the proliferative activity of the jejunal epithelium. (▲——▲), 100 mg/kg ICRF-159 at $t=0$; (●——●), 600 rad at $t=0$; (○——○), 100 mg/kg (ICRF-159) (−5 min) +600 rad ($t=0$). Each point represents the mean d/min/mg ±s.e. for 4 animals.

FIG. 2 (b).—The effect of combined fractionated ICRF-159 and 600 rad on the proliferative activity of the jejunal epithelium. (○——○), 25 mg/kg 3-hourly ×4 ICRF-159 from −12 h; (●——●), 600 rad at $t=0$; (■——■), 25 mg/kg 3-hourly ×4 ICRF-159 from −12 h + 600 rad at $t=0$. Each point represents the mean d/min/mg ±s.e. for 4 animals.

FIG. 3 (a).—Proliferation response of the LL tumour to acute and fractionated ICRF-159. (○——○), 100 mg/kg ICRF-159 at $t=0$; (●——●), 25 mg/kg, 3-hourly ×4 from −12 h. Each point represents the mean d/min/mg ±s.e. for 4 tumours. Dotted lines represent the s.e. around the mean d/min/mg for 4 untreated tumours measured throughout the course of the experiment.

FIG. 3 (b).—The effect of combined acute or fractionated ICRF-159 and radiation on the proliferative activity of the LL tumour (- - - - -), 600 rad at $t=0$; (○——○), 100 mg/kg ICRF-159 ($t=-5$ min) +600 rad ($t=0$); (●——●), 25 mg/kg 3-hourly ×4 ICRF-159 from −12 h +600 rad at $t=0$. Each point represents the mean d/min/mg ±s.e. for 4 tumours.

treatments. But more importantly, there was an enhanced proliferative overshoot after fractionated drug+radiation.

*Effect of ICRF*-159 *and X-irradiation on the proliferative activity of LL*

Unlike the jejunal response to acute and fractionated ICRF-159, the LL tumour response was markedly different for the two treatment schedules. During the 12 h of fractionated (25 mg/kg 3-hourly ×4) ICRF-159 treatment, the $^3$H-TdR incorporation gradually fell to 16% of control. Nine hours after the last drug treatment (Fig. 3a; 6 h), the $^3$H-TdR incorporation was 163% of control, returning to control levels by 24 h after treatment. Thereafter, the $^3$H-TdR levels fluctuated around the control level for 120 h. However, after an acute dose (100

mg/kg) of ICRF-159, the proliferative activity appeared relatively unchanged during the first 48 h after treatment and exceeded control levels at 96 h (Fig. 3a).

After 600 rad X-irradiation, $^3$H-TdR incorporation fell rapidly to 45% of control levels and fluctuated about the control value for 120 h (Fig. 3b). There was no evidence of compensatory proliferation following this modest radiation exposure. In fact, fractionated ICRF-159 was more effective in reducing TdR incorporation than 600 rad alone (16% *vs* 45%). Whilst the initial recovery from either acute drug (Fig. 3a) or irradiation (Fig. 3b) occurred at the same time, the initial recovery from fractionated drug took place 6 h earlier.

Both acute (100 mg/kg) and fractionated (25 mg/kg 3-hourly ×4) ICRF-159 altered the tumour response to 600 R. Combined acutely administered drug+radiation increased the depression of $^3$H-TdR incorporation over either drug (Fig. 3a) or radiation (Fig. 3b) alone. In addition, initiation of post-radiation proliferative recovery was delayed by 6 h after combined acutely administered drug+600 rad. Once recovery from the combined treatment had been initiated, it proceeded rapidly to control levels. Following combined fractionated drug+600 rad, proliferative activity remained at less than 33% of control value for 36 h and failed to return to control levels until 84 h after treatment. This prolonged depression of DNA synthesis (cell production) was in distinct contrast to what was observed either after radiation alone or after the acutely administered ICRF-159–radiation combination.

*Cytokinetic response of LL to fractionated ICRF-159+600 rad*

During the fractionated pretreatment with ICRF-159, there were changes in the cytokinetics of the LL tumour. A 10-fold increase in mitotic index (MI, Fig. 4b) accompanied the 12 h drug treatment, with a concomitant decrease in the LI (Fig. 4a), suggesting an M-phase block in

Fig. 4.—Cytokinetic response of the LL tumour to combined ICRF-159 and 600 rad. (a) % labelled interphase cells; (b) mitotic index. (○——○), 25 mg/kg 3-hourly ×4 ICRF-159 from −12 h; (▲——▲), 600 rad at $t=0$; (●——●), 25 mg/kg 3-hourly ×4 from −12 h +600 rad at $t=0$; (■) control (mean±s.e.) for 30 tumours over a period of 168 h. Every other point represents the mean±s.e. of 4 tumours.

the cell cycle. Nine hours after the last injection of drug (at −3 h) the LI rose to 150% of control, while the MI fell, suggesting a release from the M-phase block. Control values for both LI and MI were established at 12 h and remained unperturbed over the next 156 h of observation. After irradiation with 600 rad alone, the LI gradually fell to a nadir (38% of control) at 24 h, rose sharply by 48 h (133% of control), and subsequently returned to control levels by 96 h. The wave of $^3$H-TdR incorporation (d/min/mg, Fig. 3b) with no increase in LI (LI, Fig. 4a) suggests an accelerated S-phase transit time.

When fractionated ICRF-159 was combined with 600 rad ($t=0$), a rapid fall in MI to control levels by 6 h and the marked increase at 12 h differed conspicuously from the response to either drug or radiation alone (Fig. 4). In addition, the combined treatment further depressed the drug-induced reduction of the LI. This reduced LI after fractionated drug+ radiation persisted for 72 h and was more severe than with other treatments; and initiation of LI recovery was delayed. Recovery from both drug and radiation alone treatment produced an LI higher than control levels. During the recovery from combined fractionated ICRF-159+ 600 rad, the LI failed to exceed control levels.

### DISCUSSION

ICRF-159 has been reported to be both an antiproliferative and a cytotoxic agent, whose effects are dependent on both dose and drug schedule (Hellmann & Field, 1970; Sharpe *et al.*, 1970). These reports were based on results from studies *in vitro* and the cytotoxic and antiproliferative actions of the drug were found to be closely related and proliferation-dependent (Taylor & Bleehen, 1977*a*). When ICRF-159 is used with radiation in combined mode schedules, the drug–radiation interactions are extended to the critical self-renewing tissues such as the haematopoietic and intestinal epithelium, which suffer both drug and radiation insult.

Fractionated (25 mg/kg 3-hourly ×4) treatment with ICRF-159 before radiation was found to retard tumour growth significantly compared to a single dose (100 mg/kg) of the drug given 5 min before radiation (Kovacs *et al.*, 1978). Neither acute nor fractionated doses of ICRF-159 alone showed intestinal toxicity. In terms of cytokinetic effects, both treatments produced transient intestinal insults, with essentially similar recovery, the exception being that fractionated drug treatment extended the periods of antiproliferative activity. Neither pretreatment schedule, when combined with radiation, influenced the immediate recovery kinetics to 600 rad (Fig. 2). However, the maximum compensatory hyperplasia was delayed by 24 h for acute drug pretreatment, which may be the result of either the prolonged antiproliferative effect of the drug or an increase in crypt cellular damage (Lesher & Lesher, 1970; Hagemann *et al.*, 1971). Whilst other cytotoxic agents are known to modify the intestinal responses to irradiation by enhanced cell kill (Hagemann & Concannon, 1973; Schenken *et al.*, 1978; Moore & Hendry, 1978), in these studies ICRF-159, given as an acute dose before irradiation, did not enhance animal lethality or alter the proliferative response of crypt cells to X-rays. However, a fractionated drug effect appears to enhance compensatory hyperplasia. This may be attributable to antiproliferative events associated with the drug alone. Prolonged suppression of cell production without attendant cell kill may result in subsequent heightened proliferation.

Single acute doses of up to 1000 mg/kg, as well as daily doses of 25 mg/kg over a 21 day period, were tolerated by $BDF_1$ mice, and did not produce animal lethality or significant weight loss. Fractionated (25 mg/kg 3-hourly ×4) ICRF-159 was also well tolerated; however, when a dose of 5 mg/kg was delivered q 4h × 6–9, animal mortality approached 100% within 6 days after treatment had begun (Schenken, unpublished).

The differences between acute and fractionated ICRF-159 on proliferation are more obvious in the LL tumour than in the intestinal epithelium. Both fractionated and acute doses of ICRF-159 reduce the proliferative activity of the intestinal mucosa to nearly the same level (Fig. 1), whereas fractionated ICRF-159 treatment is far more effective in depressing tumour proliferation than an equivalent acute dose of the drug (Fig. 3a). Dose/time differentiated effects for both the cytotoxic and cytostatic actions of ICRF-159 on the EMT6 cell line *in vitro* have been reported (Taylor & Bleehen, 1977*a*). Creighton & Birne (1970), using mouse embryo cultures incubated with ICRF-159 for 22 h, found a rapid inhibition of $^3$H-TdR incorporation into DNA increasing with concentration up to 2 $\mu$g/ml. Thereafter, much greater increases in drug concentration were required to produce additional inhibition of

DNA synthesis. From the results presented here (Figs. 3a and 3b) the time courses of responses to radiation or acute treatment with ICRF-159 are similar, but very different from fractionated drug treatment. During the protracted treatment with ICRF-159, proliferative activity in LL is depressed (Fig. 3b), primarily owing to an accumulation of cells in mitosis (Fig. 4b) with a concomitant decrease in the S-phase compartment size (Fig. 4a). This is in distinct contrast to an earlier suggestion that ICRF-159 blocked the transit of cells from $G_2$ into mitosis (Sharpe *et al.*, 1970).

Hallowes *et al.* (1974) have observed that the effect of exposure to the drug is reversible, provided a threshold-level concentration is not maintained in the medium. With repeated exposures, the drug effect was cumulative, resulting in an inhibition of cell division with continued DNA synthesis. Field *et al.* (1971) reported a rapid, exponential clearance rate of ICRF-159 from rat blood with a half-time of 25 min. If the clearance rate for ICRF-159 is similarly rapid in the mouse, treatment at 3h intervals with 25 mg/kg may not maintain a high enough concentration of the drug for long enough to establish an irreversible block in $G_2$. Rather, the transit of cells through $G_2$ may be serially interrupted with successive treatments, generating increments in the mitotic index and subsequent synchronization.

Combined treatment with ICRF-159 and radiation produces an enhanced antiproliferative effect in LL. Although most radiopotentiating effects of ICRF-159 have been attributed to a drug-induced angiometamorphic effect and its consequences, rather than to an inhibition of repair of radiation damage (Hellmann & Murkin, 1974), Taylor & Bleehen (1977*a*) have suggested that ICRF-159 substantially decreases the width of the shoulder of the radiation-response curve. However, they suggest that ICRF-159 reduced the ability of drug-treated cells to accumulate sublethal damage rather than prevented the repair of such damage (Taylor & Bleehen, 1977*b*). Also, from the data presented herein and by Peters (1975), there is evidence of a partial redistribution of tumour cells into a more sensitive state. The radiation response of a population of cells depends to a great extent on both the age-response function and the age-density distribution of the population. We have shown that fractionated ICRF-159 treatment alters the age-density distribution of the cells within LL solid tumours.

From these studies it seems clear that the differences between fractionated and acute ICRF-159 in combined-mode approaches are closely linked to the differences in the proliferative responses to the drug. Both concentration and duration are determinants which have been considered for the effective use of drugs in combined-model therapy (*i.e.*, drug–radiation, drug–drug) to prevent toxicities. The results described here suggest that low-dose, fractionated treatment with ICRF-159 for 12 h enhances the radiation response of the tumour, but does not enhance intestinal toxicity. We are presently investigating other ICRF-159 fractionation schedules in a number of combined-mode sequences.

This investigation was supported by Grant Numbers RO1 CA20328 and CA10438, awarded by the National Cancer Institute, DHEW.

The authors wish to express their appreciation to Dr H. A. Hopkins for reading the manuscript and to Ms C. Mallick and L. Keller for their technical assistance.

### REFERENCES

Bellet, R. E., Mastrangelo, J. J., Dixon, L. M. & Yarbo, J. W. (1973) Phase I study of ICRF-159 (NSC-129943) in human tumors. *Cancer Chemother. Rep.*, **57**, 185.

Creighton, A. M. & Birne, G. D. (1970) Biochemical studies on growth-inhibitory bisdioxopiperazines. I. Effect on DNA, RNA and protein synthesis in mouse-embryo fibroblasts. *Int. J. Cancer*, **5**, 47.

Field, E. O., Mauro, F. & Hellmann, K. (1971) Blood clearance of ICRF-159 (NSC-129943). *Cancer Chemother. Rep.*, **55**, 527.

Gralla, E. J., Coleman, G. L. & Jones, A. M. (1974) Preclinical toxicology studies with ICRF-159 (NSC-129943)—a new antineoplastic drug. *Cancer Chemother. Rep.*, **58**, 1.

Hagemann, R. F. & Concannon, J. P. (1973) Mechanism of intestinal radiosensitization by actinomycin D. *Br. J. Radiol.*, **46**, 302.

HAGEMANN, R. F., SIGDESTAD, C. P. & LESHER, S. (1971) Intestinal crypt survival and total and per crypt levels of proliferative cellularity following irradiation: single X-ray exposures. *Radiat. Res.*, **46**, 533.

HALLOWES, R. C., WEST, D. G. & HELLMANN, K. (1974) Cumulative cytostatic effect of ICRF-159. *Nature*, **247**, 487.

HELLMANN, K. & FIELD, E. O. (1970) Effect of ICRF-159 on the mammalian cell cycle: significance for its use in cancer chemotherapy. *J. Natl Cancer Inst.*, **44**, 539.

HELLMANN, K. & MURKIN, G. E. (1974) Synergism of ICRF-159 and radiotherapy in treatment of experimental tumors. *Cancer*, **34**, 1033.

KOVACS, C. J., EVANS, M. J., SCHENKEN, L. L. & BURHOLT, D. R. (1979) ICRF-159-induced enhancement of radiation response in combined modality therapies. I. Time/dose relationships for tumour response. *Br. J. Cancer*, **39**, 516.

LESHER, J. & LESHER, S. (1970) Effects of single-dose, whole-body, $^{60}Co$ gamma irradiation on number of cells in DNA synthesis and mitosis in the mouse duodenal epithelium. *Radiat. Res.*, **43**, 429.

MOORE, J. V. & HENDRY, J. H. (1979) Response of murine jejunal crypts to single doses of cyclophosphamide and radiation. *Int. J. Radiat. Oncol., Biol. Phys.* (in press).

NORPOTH, K., SCHAPHAUS, A., ZIEGLER, H. & WITTING, V. (1974) Combined treatment of the Walker tumour with radiotherapy and ICRF-159. *Z. Krebsforsch.*, **82**, 324.

PETERS, L. J. (1975) Modification of the radiocurability of a syngeneic murine squamous carcinoma by its site of growth, by electron-affinic drugs, and by ICRF-159. *Br. J. Radiol.*, **49**, 708.

RYALL, R. D. H., HARHAM, I. W. F., NEWTON, K. A., HELLMANN, K., BRINKLEY, D. & HJERTAAS, O. K. (1974) Combined treatment of soft tissue and osteosarcomas by radiation and ICRF-159. *Cancer*, **34**, 1040.

SCHENKEN, L. L. (1976) Proliferative character and growth modes of neoplastic disease as determinants of chemotherapeutic efficacy. *Cancer Treat. Rep.*, **60**, 1761.

SCHENKEN, L. L., BURHOLT, D. R., HAGEMANN, R. F. & KOVACS, C. J. (1978) Combined modality oncotherapies: cell kinetic approaches for avoidance of gastrointestinal toxicity. In *Frontiers of Radiation Therapy and Oncology*. Ed. J. M. Vaeth. Basel: Karger. p. 82.

SHARPE, H. B. A., FIELD, E. O. & HELLMANN, K. (1970) Mode of action of the cytostatic agent ·ICRF-159'. *Nature*, **226**, 524.

TAYLOR, I. W. & BLEEHEN, N. M. (1977*a*) Changes in sensitivity to radiation and ICRF-159 during the life of monolayer cultures of EMT6 tumour lines. *Br. J. Cancer*, **35**, 587.

TAYLOR, I. W. & BLEEHEN, N. M. (1977*b*) Interaction of ICRF-159 with radiation, and its effect on sublethal and potentially lethal radiation damage *in vitro*. *Br. J. Cancer*, **36**, 493.


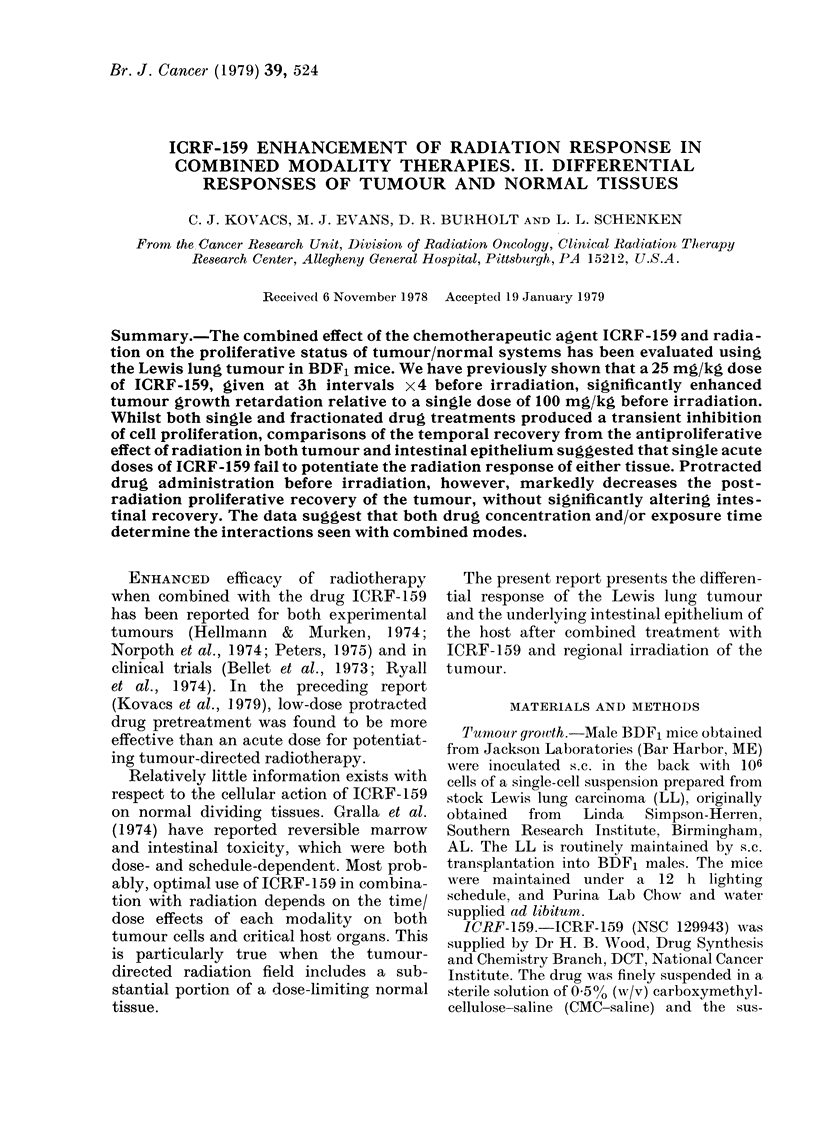

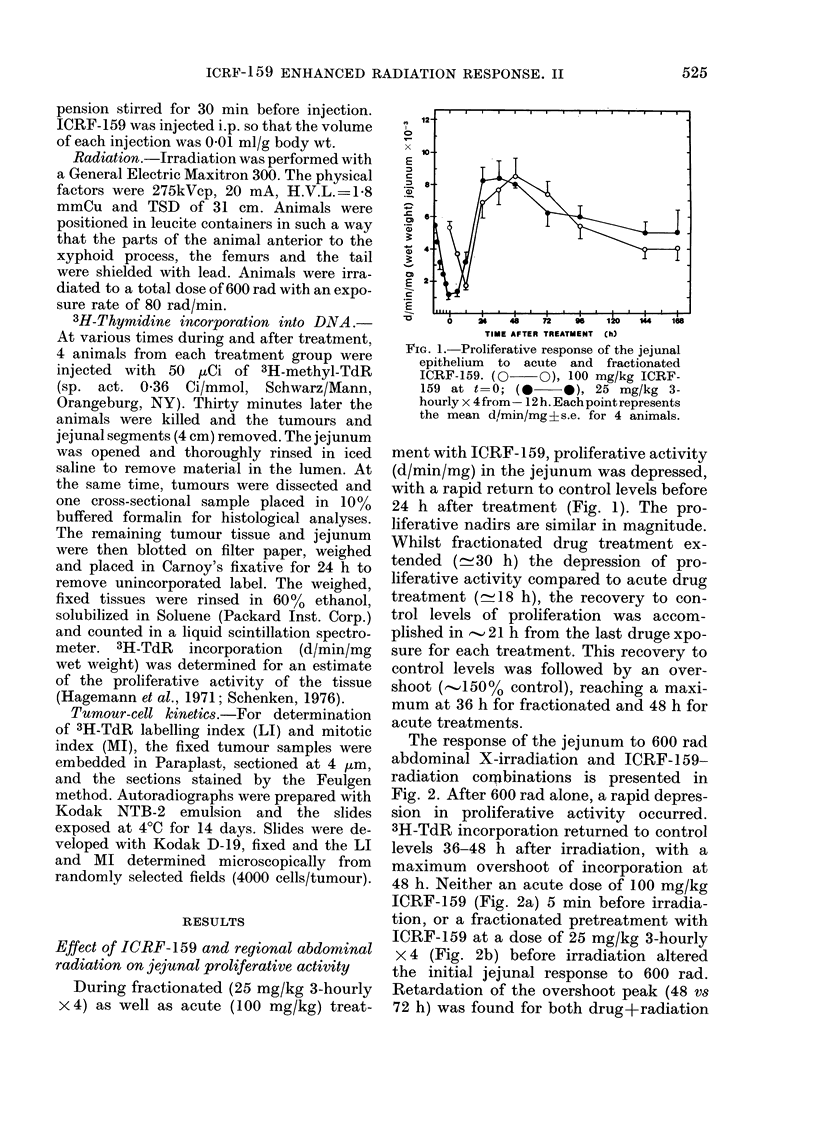

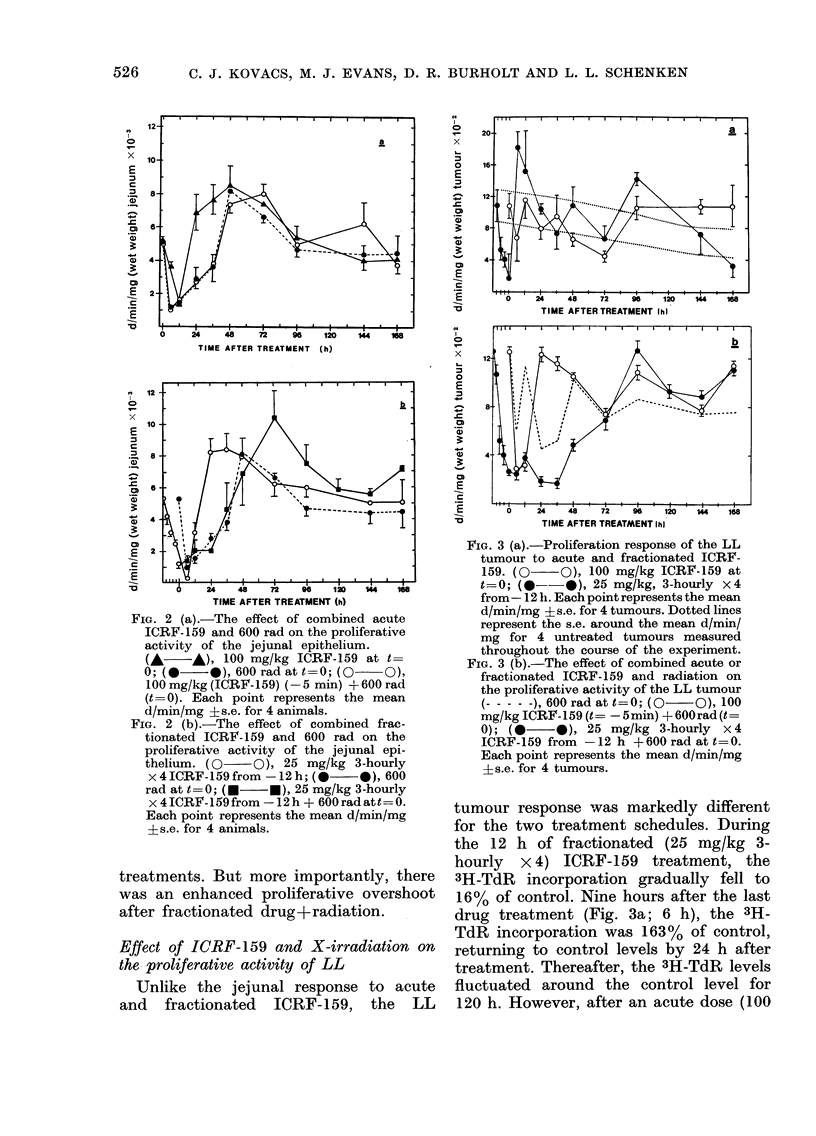

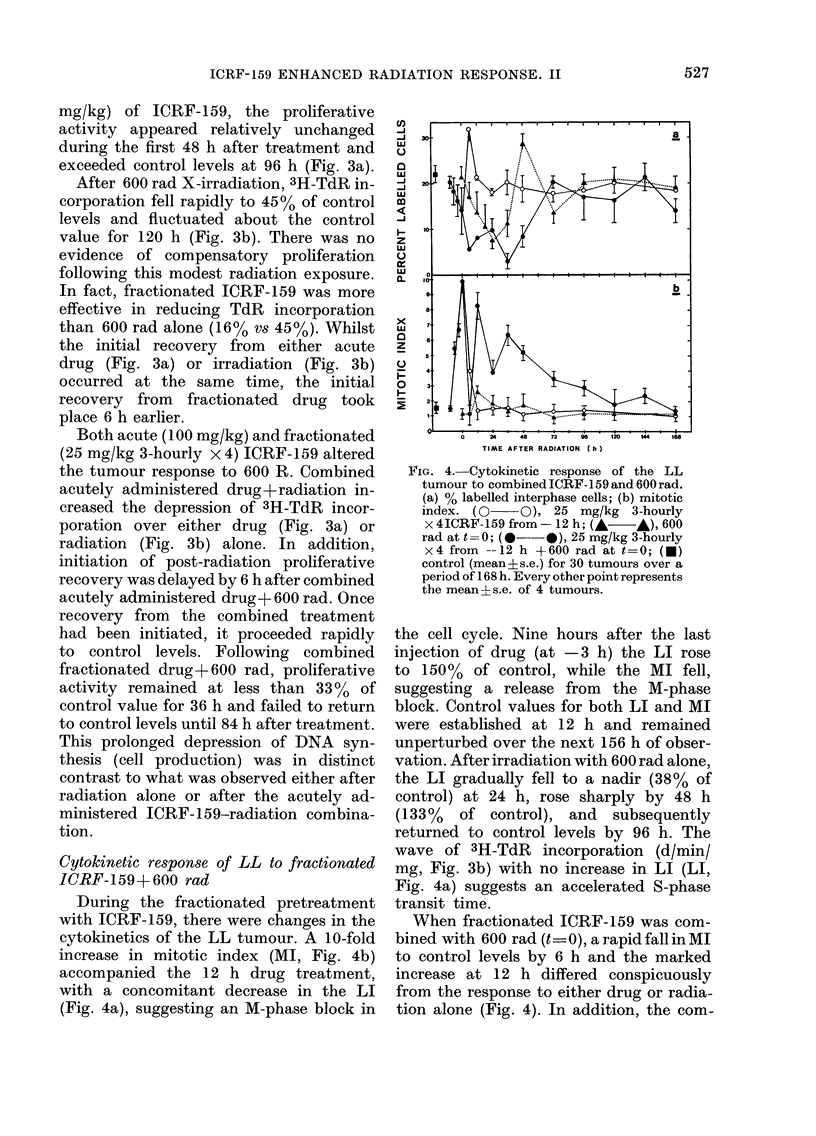

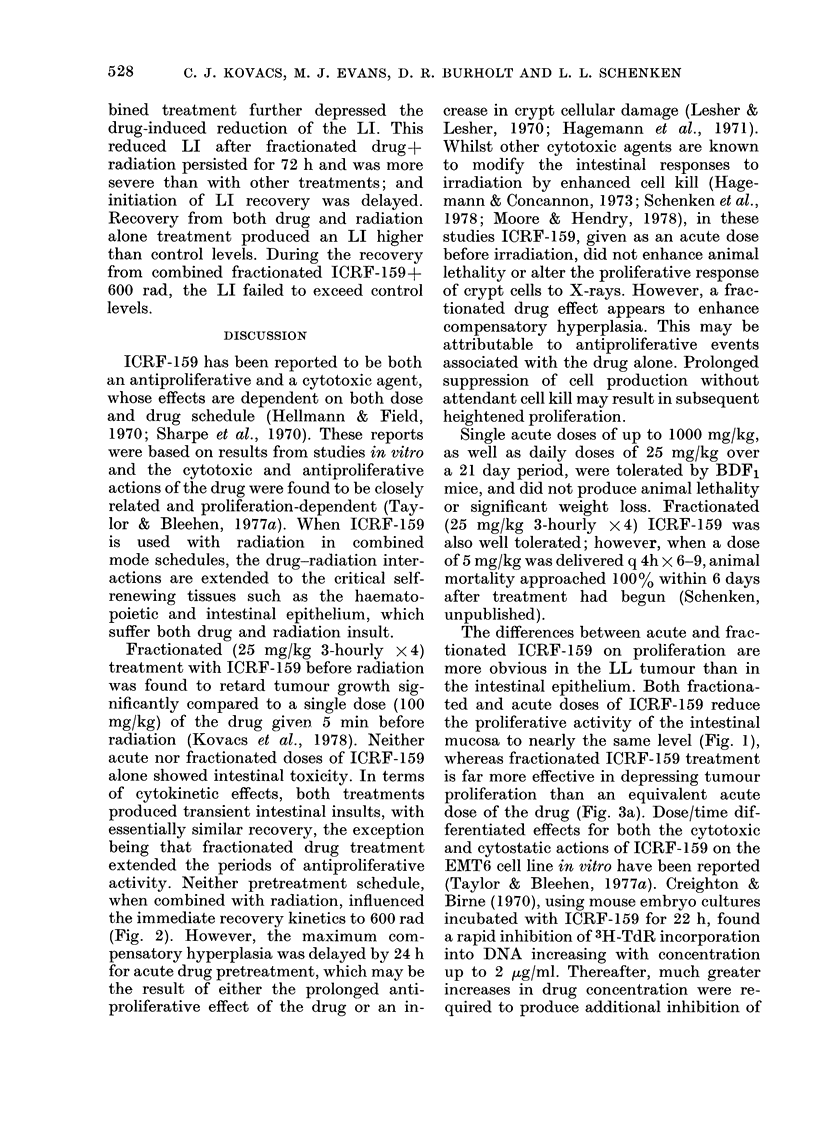

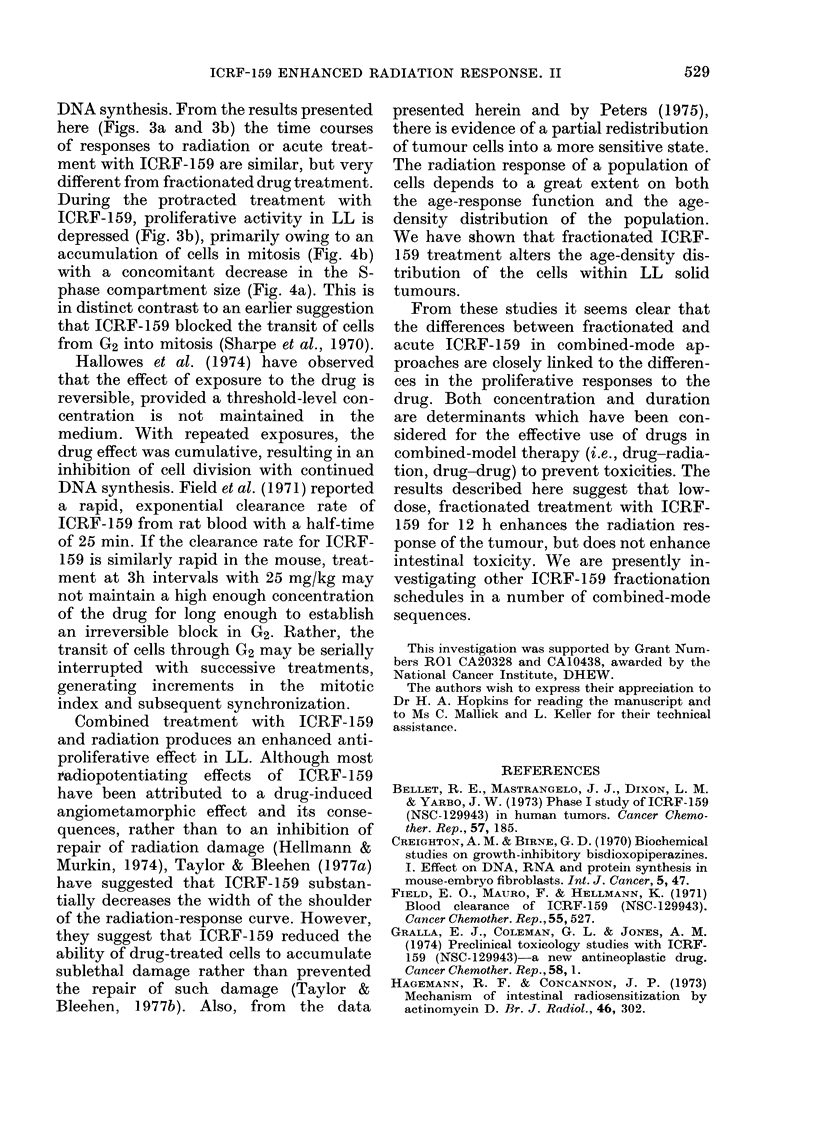

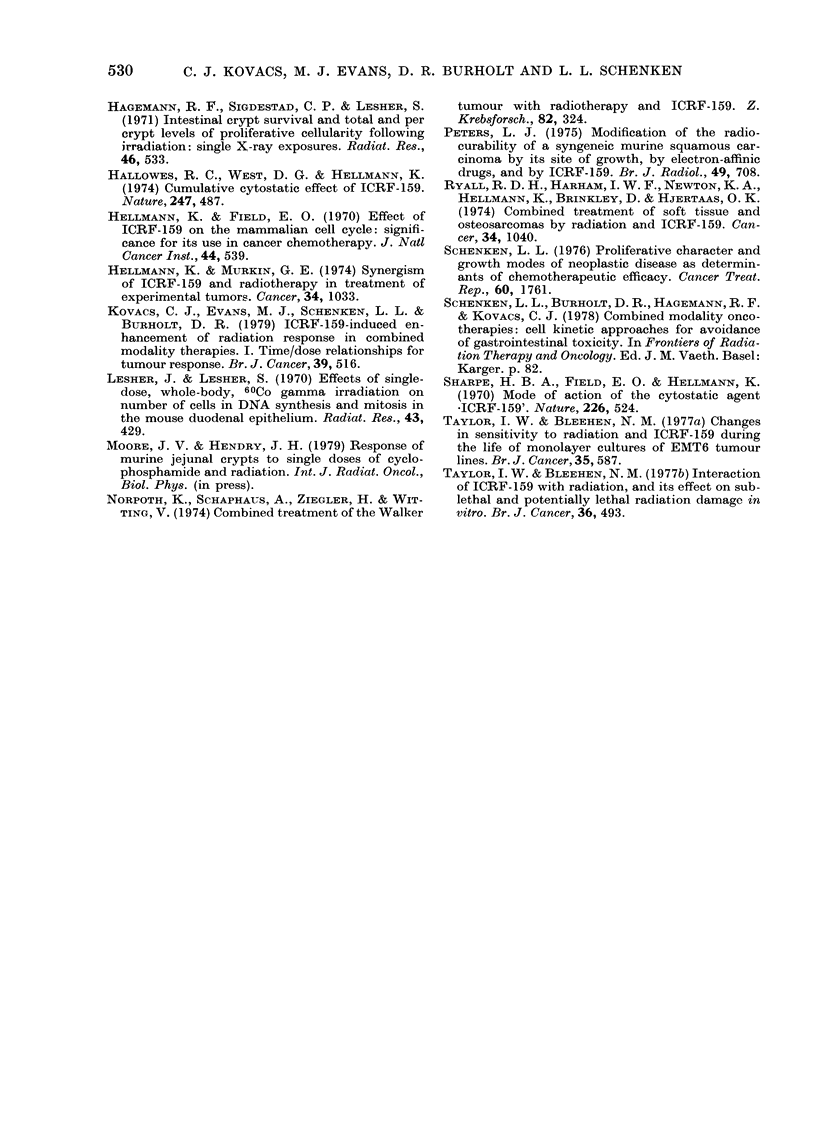


## References

[OCR_00736] Bellet R. E., Mastrangelo M. J., Dixon L. M., Yarbro J. W. (1973). Phase I study of ICRF-159 (NSC-129943) in human solid tumors.. Cancer Chemother Rep.

[OCR_00742] Creighton A. M., Birnie G. D. (1970). Biochemical studies on growth-inhibitory bisdioxopiperazines. I. Effect on DNA, RNA and protein synthesis in mouse-embryo fibroblasts.. Int J Cancer.

[OCR_00748] Field E. O., Mauro F., Hellmann K. (1971). Blood clearance of ICRF 159 (NSC-129943).. Cancer Chemother Rep.

[OCR_00753] Gralla E. J., Coleman G. L., Jonas A. M. (1974). Preclinical toxicology studies with ICRF-159 (NSC-129943)--a new antineoplastic drug.. Cancer Chemother Rep 3.

[OCR_00759] Hagemann R. F., Concannon J. P. (1973). Mechanism of intestinal radiosensitization by actinomycin D.. Br J Radiol.

[OCR_00766] Hagemann R. F., Sigdestad C. P., Lesher S. (1971). Intestinal crypt survival and total and per crypt levels of proliferative cellularity following irradiation: single x-ray exposures.. Radiat Res.

[OCR_00773] Hallowes R. C., West D. G., Hellmann K. (1974). Cumulative cytostatic effect of ICRF 159.. Nature.

[OCR_00778] Hellmann K., Field E. O. (1970). Effect of ICRF159 on the mammalian cell cycle: significance for its use in cancer chemotherapy.. J Natl Cancer Inst.

[OCR_00784] Hellmann K., Murkin G. E. (1974). Synergism of ICRF 159 and radiotherapy in treatment of experimental tumors.. Cancer.

[OCR_00789] Kovacs C. J., Evans M. J., Schenken L. L., Burholt D. R. (1979). ICRF-159 enhancement of radiation response in combined modality therapies. I. Time/dose relationships for tumour response.. Br J Cancer.

[OCR_00796] Lesher J., Lesher S. (1970). Effects of single-dose, whole-body, 60Co gamma irradiation on number of cells in DNA synthesis and mitosis in the mouse duodenal epithelium.. Radiat Res.

[OCR_00816] Peters L. J. (1976). Modification of the radiocurability of a syngeneic murine squamous carcinoma by its site of growth, by electron-affinic drugs, and by ICRF 159.. Br J Radiol.

[OCR_00821] Ryall R. D., Hanham I. W., Newton K. A., Hellmann K., Brinkley D. M., Hjertaas O. K. (1974). Combined treatment of soft tissue and osteosarcomas by radiation and ICRF 159.. Cancer.

[OCR_00828] Schenken L. L. (1976). Proliferative character and growth modes of neoplastic disease as determinants of chemotherapeutic efficacy.. Cancer Treat Rep.

[OCR_00842] Sharpe H. B., Field E. O., Hellmann K. (1970). Mode of action of the cytostatic agent "ICRF 159".. Nature.

[OCR_00847] Taylor I. W., Bleehen N. M. (1977). Changes in sensitivity to radiation and ICRF 159 during the life of monolayer cultures of EMT6 tumour line.. Br J Cancer.

[OCR_00853] Taylor I. W., Bleehen N. M. (1977). Interaction of ICRF 159 with radiation, and its effect on sub-lethal and potentially lethal radiation damage in vitro.. Br J Cancer.

